# Animal Physiology. (From the Popular Cyclopædia of Natural Science.)

**Published:** 1844-01

**Authors:** 


Art. I.—*Animal Physiology.* (*From the Popular Cyclopædia of Natural Science.*) By W. B. Carpenter, m.d. &c.—*London*, 1843. 8vo, pp. 580.

This work, which forms part of the 'Popular Cyclopædia of Natural Science,' like all the writings of the author, affords a clear exposition of the subject of which it treats, and is well calculated to render the general reader familiar with some of the most abstruse, but most important, subjects of physiology. It is written in a familiar and exceedingly clear style, and is, we think, calculated to convey most valuable information not only to the general reader but also to the medical student. The plan of combining natural history with physiology is the only way in which this latter subject can ever be made popular. To illustrate abstruse questions by the familiar example of some interesting and constantly occurring facts in natural history, not only tends to lighten scientific details, but puts the facts themselves in a new position, and gives additional force to them. This has been managed by Dr. Carpenter in the present volume in a very admirable and pleasing manner. We may point to some of the chapters in proof of our remark. Take for instance the chapter on the Circulatory System. The whole of this subject is well worked-out up to the period when this part of the 'Cyclopædia' was published. We do not hesitate to affirm that while this will afford a vast deal of information to the general reader, it will convey much new information not merely to medical students but to medical practitioners generally. So again, in regard to the Functions of the Nervous System. This chapter gives a more precise and definite notion of the functions of this part of the body than any book we have yet met with. Again, the chapter on Reproduction and Development will convey an excellent idea of these great functions, without obliging the reader to wade through details that would often tend to fatigue, and perhaps to create some displeasure with the subject in the minds of non-professional readers, for whom these volumes have been expressly written. But, in truth, the whole work is of that genuine masterly stamp which characterizes all the productions of this writer, even those, such as we believe this to be, which are the fruits of his leisure hours, and are not intended for the learned. It is certainly without parallel among the treatises on subjects of natural science hitherto presented to the public, and may be confidently recommended to readers of every class, as fraught with important information not elsewhere to be found in so agreeable a form.

